# Novel insight into the genetic signatures of altitude adaptation related body composition in Tibetans

**DOI:** 10.3389/fpubh.2024.1355659

**Published:** 2024-05-14

**Authors:** Xuguang Li, Shilin Xu, Xuemei Li, Yuhe Wang, Yemeng Sheng, Hengxun Zhang, Wei Yang, Dongya Yuan, Tianbo Jin, Xue He

**Affiliations:** ^1^Key Laboratory of Molecular Mechanism and Intervention Research for Plateau Diseases of Tibet Autonomous Region, School of Medicine, Xizang Minzu University, Xianyang, Shaanxi, China; ^2^Key Laboratory of High Altitude Hypoxia Environment and Life Health, School of Medicine, Xizang Minzu University, Xianyang, Shaanxi, China; ^3^Department of Clinical Laboratory, The Affiliated Hospital of Xizang Minzu University, Xianyang, Shaanxi, China; ^4^Department of Healthcare, The Affiliated Hospital of Xizang Minzu University, Xianyang, Shaanxi, China; ^5^Department of Emergency, The Affiliated Hospital of Xizang Minzu University, Xianyang, Shaanxi, China; ^6^School of Medicine, Xizang Minzu University, Xianyang, Shaanxi, China

**Keywords:** GWAS, high altitude adaptation, body compositions, Tibetan college students, *EPAS1*

## Abstract

**Background:**

The Tibetan population residing in high-altitude (HA) regions has adapted to extreme hypoxic environments. However, there is limited understanding of the genetic basis of body compositions in Tibetan population adapted to HA.

**Methods:**

We performed a genome-wide association study (GWAS) to identify genetic variants associated with HA and HA-related body composition traits. A total of 755,731 single nucleotide polymorphisms (SNPs) were genotyped using the precision medicine diversity array from 996 Tibetan college students. T-tests and Pearson correlation analysis were used to estimate the association between body compositions and altitude. The mixed linear regression identified the SNPs significantly associated with HA and HA-related body compositions. LASSO regression was used to screen for important SNPs in HA and body compositions.

**Results:**

Significant differences were observed in lean body mass (LBW), muscle mass (MM), total body water (TBW), standard weight (SBW), basal metabolic rate (BMR), total protein (TP), and total inorganic salt (Is) in different altitudes stratification. We identified three SNPs in *EPAS1* (rs1562453, rs7589621 and rs7583392) that were significantly associated with HA (*p* < 5 × 10^−7^). GWAS analysis of 7 HA-related body composition traits, we identified 14 SNPs for LBM, 11 SNPs for TBW, 15 SNPs for MM, 16 SNPs for SBW, 9 SNPs for BMR, 12 SNPs for TP, and 26 SNPs for Is (*p* < 5.0 × 10^−5^).

**Conclusion:**

These findings provide insight into the genetic basis of body composition in Tibetan college students adapted to HA, and lay the foundation for further investigation into the molecular mechanisms underlying HA adaptation.

## 1 Introduction

High altitude (HA), which is defined as being above 2,500 m (approximately 8,200 feet) above sea level (mASL), is typically characterized by low atmospheric pressure, thin oxygen levels, and low temperatures. Short-term exposure to HA anoxic environments could lead to acute altitude sickness, with symptoms including high altitude pulmonary edema (HAPE) and cerebral edema (1). Long-term exposure to these environments can also increase the risk of pregnancy complications, such as preeclampsia (2). However, genetic adaptation to a new environment is a fundamental process of species survival and adaptation. Populations that have resided at HA for generations have experienced selective pressures and undergone physiological and genetic adaptations to thrive in anoxic environments. Exposure to hypoxia enables an animal’s homeostasis system to effectively respond to changes in oxygen concentration, which is crucial for survival (3). Tibetans have lived at very HA for thousands of years, and they possess distinctive physiological traits that enable them to adapt to the HA environment (4).

Body composition refers to the composition of various components, such as water, muscle, fat, and inorganic salts in the human body, as well as their percentage of the total body mass. The composition of the body is a crucial factor that impacts human health and has gained increasing attention in recent years. Systematic review and meta-analysis studies have reported significant reductions in body weight, fat mass (FM), fat free mass (FFM), and lean body mass (LBM) of individuals exposed to HA (5, 6). In the healthy indigenous populations living on the Qinghai-Tibet Plateau, protein mass, bone mass (BM), FM and body water values decrease with increasing altitude (7). Sympathetic activity is reduced during prolonged exposure to HA, resulting in a decrease in basal metabolic rate (BMR) (6). Additionally, exposure to HA hypoxic environment can lead to serious loss of muscle mass (MM), which results in skeletal muscle atrophy (8). Participants living at sea level tend to be taller, heavier, and have a higher body mass index (BMI), and waist circumference (WC) relative to those living at HA (9). Previous studies have shown that the growth indicators, such as height, weight, chest circumference, and WC, differ among Chinese Tibetan adolescents at different altitudes (10). However, there is a lack of systematic research on the differences in human body composition among Tibetan college students at different altitudes. Therefore, understanding the differences in body composition among Tibetan populations at different altitudes can provide valuable insights into the physiological mechanisms of HA adaptation.

Over the past decade, numerous studies and more recent genomic association analysis studies have provided evidence for the genetic basis of these physiological changes (11, 12). A large-scale genome-wide study (GWAS) was conducted to identify genetic signals of HA adaptation at nine genomic loci, seven of which are unique to 3,008 Tibetans and 7,287 non-Tibetan individuals of Eastern Asian ancestry (13). Additionally, a significant number of GWAS have been conducted on various populations, exploring body components such as energy expenditure, LBM, WC, waist-hip ratio (WHR), height, BMI, and body fat have been widely reported (14–19). However, there have been no reports on the systematic GWASs of body composition in Tibetan college students.

In this study, we aim to conduct a GWAS on 996 Tibetan college students from different HA areas in Tibet to identify genetic variants associated with HA and HA-related body components indicators, including body fat ratio (BFR), body fat, LBM, total body water (TBW); MM, BMI, obesity, standard weight (SBW), WHR, BMR, total energy expenditure (TEE), impedance (IM), total protein (TP), and total inorganic salt (Is). These findings will contribute to a deeper understanding the genetic basis of body composition of Tibetan college students and reveal the intricate molecular mechanisms of HA adaptation.

## 2 Materials and methods

### 2.1 Participants

**Figure 1:**
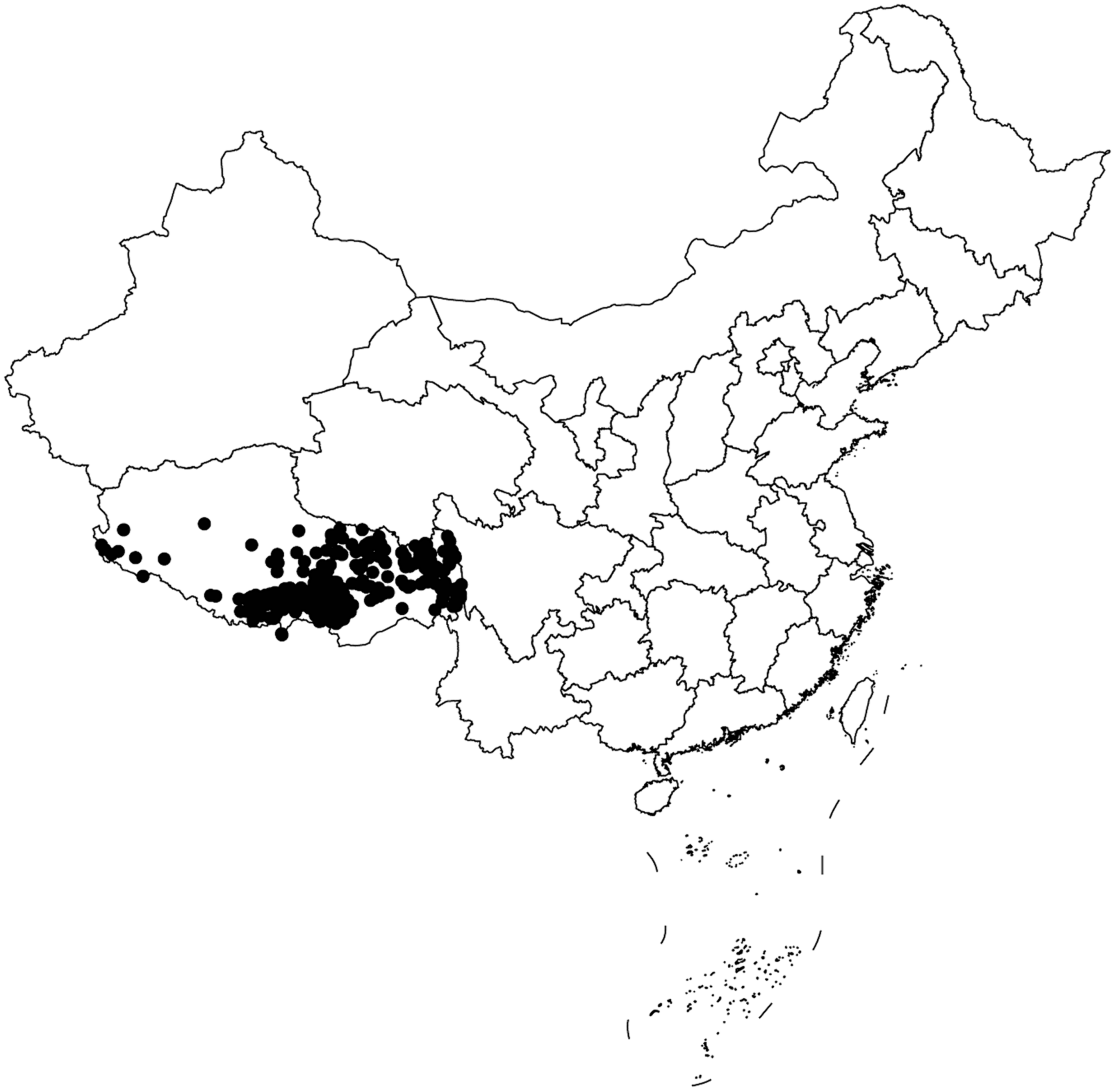
Scatter plot of samples distribution.

This study recruited a total of 996 students (545 females and 451 males, aged 16–25 years) from Tibetan freshmen in the classes 2019 and 2020 at Xizang Minzu University. All participants were healthy Tibetan college students from different altitudes in Tibet, and all participants had lived in the region for at least three generations (Figure 1). The demographic information of the research subjects, including gender, age, population, residential history, etc., was collected through a questionnaire survey. We then used websites to check the altitude,^1^ air pressure,^2^ latitude, and longitude^3^ of the participants’ habitual residence. Participants whose habitual residence was below 1,500 meters or above 5,500 meters above sea level were excluded. Peripheral venous blood sample (5 mL) was collected from each subject using EDTA anticoagulant tubes and stored in a refrigerator at-80°C for future use. This study was approved by the Ethics Committee of the Medical College of Xizang Minzu University (No. 20180–18) and was conducted in accordance with the Declaration of Helsinki. All participants signed written informed consent.

### 2.2 Body composition traits detection

All subjects’ weight, height, blood pressure and WC were measured on an empty stomach in the morning. The InBody720 body composition analyzer was used to measure various body composition indicators, including BMI, BFR, body fat, LBM, TBW, MM, obesity, SBW, WHR, BMR, TEE, IM, TP, and Is.

### 2.3 Genotyping and quality control

DNA was extracted from peripheral blood using the GoldMag DNA Extraction Kit (GoldMag Co. Ltd.). The concentration and purity of DNA were subsequently determined using a NanoDrop 2000 spectrophotometer (Thermo Scientific). The Precision medicine diversity array (PMDA) high-throughput chip was processed using GeneTitan multichannel instruments, and the extracted genomic DNA samples were genotyped. All genotyping data obtained were analyzed using Applied Biosystems™ Axiom™ software. A total of 755,731 single nucleotide polymorphisms (SNPs) were genotyped in 996 participants. A total of 476,123 of the SNPs were removed for one or more of the following standards: call rate < 90%; Hardy–Weinberg equilibrium (HWE) < 1.0 × 10^−6^; minor allele frequency (MAF) < 0.05. After imposing these constraints, 996 individuals and 279, 608 SNPs were used for statistical analyses.

### 2.4 LASSO deep learning algorithm screening HA associated SNPs

The least absolute shrinkage and selection operator (LASSO) is a regression-based approach that incorporates penalty to estimate regression coefficients. It achieves this by maximizing the logarithmic likelihood function. The main concept behind LASSO is to minimize the residual sum of squares while putting a constraint on the sum of the absolute values of the regression coefficients, ensuring it is less than a constant. This constraint enables the identification of regression coefficients that are strictly equal to zero, resulting in optimal screening results. In our study, all SNPs variables were converted into independent variables, while HA and body composition traits served as dependent variables. To determine the appropriate adjustment parameter (λ) for LASSO logistic regression, we utilized the internal validation method of 10-fold cross-validation with the minimum criterion and the 1-SE of the minimum criterion.

### 2.5 Statistical analysis

After organizing the data using Microsoft Excel, statistical processing was performed using SPSS 25.0 statistical software. The measurement data exhibited normally distributed, and the results were expressed as mean ± standard deviation (X ± S). One-way analysis of variance (ANOVA) was used to evaluate the statistical significance among multiple groups. Pearson correlation analysis was employed to determine the extent of direct and indirect influence between body composition change and altitude. The correlation coefficient is a real number ranging from −1 to +1. A correlation coefficient (*r*) between-1 and 0, with a *p*-value <0.05, indicates a negative correlation between variables. Conversely, a correlation coefficient between 0 and 1, suggests a positive correlation between variables. If *p* > 0.05, there was no statistically significant correlation between the variables. We used mixed linear regression under an additive genetic model in Gold Helix SNP & Variation Suite software (version 8.7) to identify SNPs significantly associated with HA and body composition traits. The threshold for significance was set at *p* < 5.0 × 10^−5^. Manhattan plots were constructed to visualize the genome-wide association results for altitude and body composition traits. Quantile–quantile (Q–Q) plots were used to assess the validity of the distributional assumption for the dataset. Additionally, the genomic inflation factor (λ) was calculated to compare the distribution of the test statistics across the genome with the expected null distribution. Regional plots for top SNPs were created using Locus Zoom.^4^

## 3 Results

### 3.1 Descriptive characteristic of the subjects

**Figure 2:**
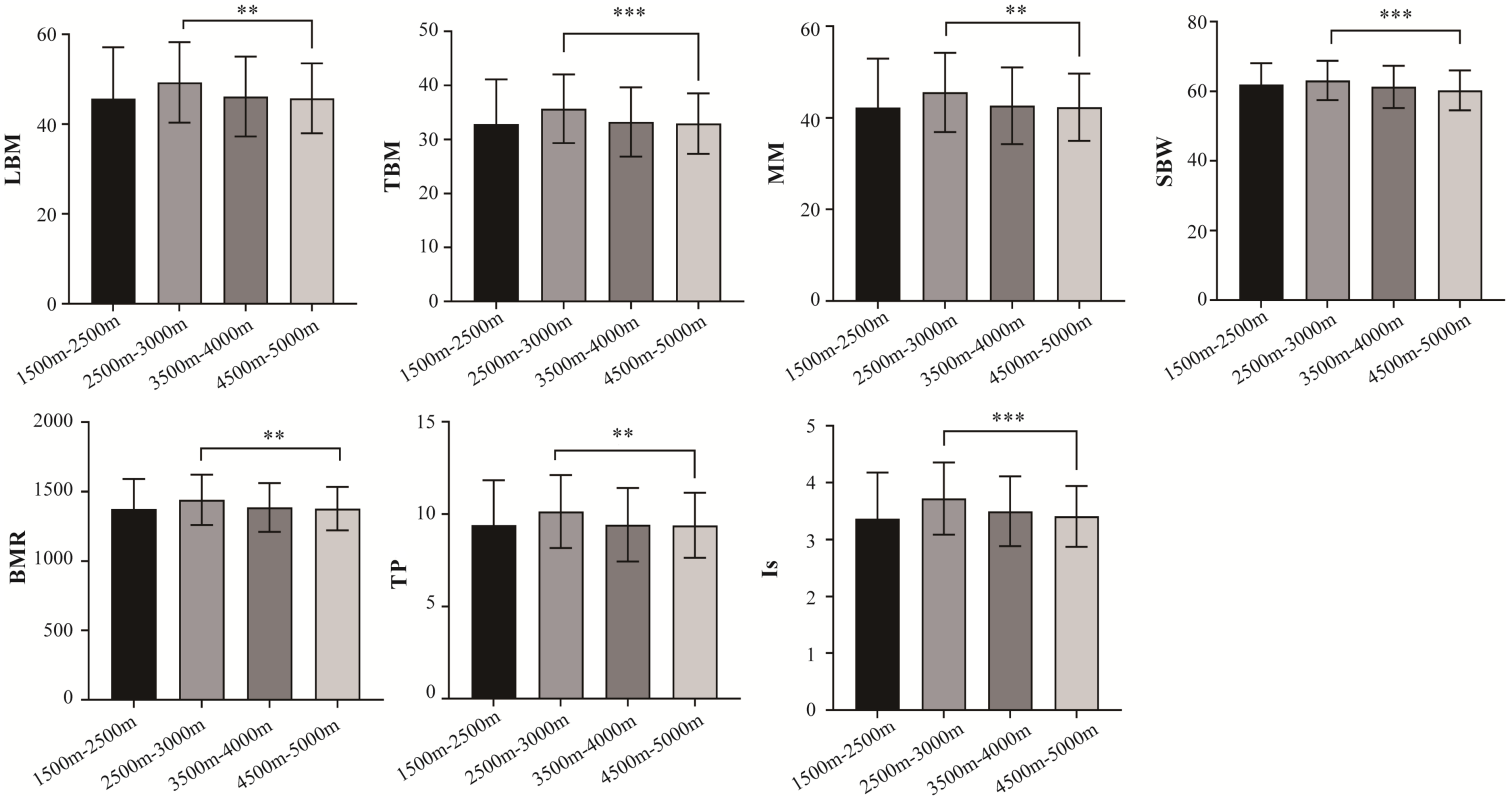
Comparison of body composition traits between different altitudes. LBM, lean body mass; TBW; total Body Water; SBW, standard weight; BMR, basal metabolic rate; IM, impedance; TP, total protein; protein; IS, inorganic salt. ^*^p < 0.05, ^**^p < 0.01, ^***^p < 0.001.

A total of 996 Tibetan college students were included in this study. Among them, 2 were excluded from the study due to residing at elevations below 1,500 m and above 5,500 m. The included participants consisted of 449 male students (45.17%) and 545 female students (54.83%). The total number of samples collected for human composition indicators can be found in Supplementary Table S1. Table 1 depicts the mean ± standard deviation values of 14 human body component indicators across different altitude stratifications. Figure 2 displays the *T*-tests results comparing different altitude groups. The results indicated significant differences (*p* < 0.05) in LMB, TBW, MM, SBW, BMR, TP, and Is between the altitude stratifications of 2,500–3,500 m and 4,500–5,500 m. Notably, LBM, TBW, MM, SBW, BMR, TP, and Is were highest at altitudes between 2,500–3,500 m above sea level and tended to decrease with increasing altitude.

**Table 1:** Description of human body composition indicators in different altitude stratification.

Variables	1,500–2,500 m	2,500–3,500 m	3,500–4,500 m	4,500–5,500 m	*p*-value
BFR (%)	21.93 ± 6.33	22.77 ± 8.21	23.78 ± 8.4	22.4 ± 8.37	0.223
BFT (kg)	12.98 ± 5.42	14.87 ± 6.96	14.56 ± 6.38	13.41 ± 6.31	0.191
LBM (kg)	45.63 ± 11.47	49.28 ± 8.96	46.14 ± 8.89	45.74 ± 7.78	**0.005**
TBW (L)	32.86 ± 8.27	35.7 ± 6.36	33.23 ± 6.41	32.93 ± 5.6	**0.002**
Muscle mass (kg)	42.26 ± 10.7	45.56 ± 8.67	42.66 ± 8.39	42.33 ± 7.35	**0.007**
BMI (kg/m2)	20.73 ± 4.13	22.32 ± 3.34	21.69 ± 3.08	21.55 ± 3.06	0.137
Obesity	−5.72 ± 18.8	1.92 ± 15.35	−0.96 ± 14.07	−1.75 ± 13.99	0.119
SBW (kg)	61.88 ± 6.15	63.11 ± 5.66	61.26 ± 6.06	60.27 ± 5.74	**0.003**
WHR	0.75 ± 0.06	0.77 ± 0.06	0.77 ± 0.06	0.77 ± 0.06	0.679
BMR (kcal/day)	1374.64 ± 216.84	1441.23 ± 181.21	1386.28 ± 176.24	1378.46 ± 156.12	**0.018**
TEE (kcal/day)	2057.73 ± 319.8	2123.27 ± 301.48	2050.45 ± 298.5	2056.3 ± 271.19	0.131
IM (Ω)	554.73 ± 110.38	498.82 ± 83.56	522.1 ± 83.51	509.87 ± 73.11	**0.012**
TP (g/dL)	9.4 ± 2.44	10.14 ± 1.97	9.43 ± 2	9.4 ± 1.77	**0.006**
Is (g)	3.36 ± 0.82	3.72 ± 0.63	3.5 ± 0.61	3.41 ± 0.53	**0.001**

BFR, body fat ratio; BFT, body fat; LBM, lean body mass; TBW, total body water; BMI, body mass index; SBW, standard weight; WHR, waist to hip ratio; BMR, basal metabolic rate; TEE, total energy expenditure; IM, impedance; TP, total protein; Is, inorganic salt. *p-*values were calculated from Student’s *t-*test (two-sided). Bold font and *p* < 0.05 indicates statistical significance.

### 3.2 SNPs associated with HA

**Figure 3:**
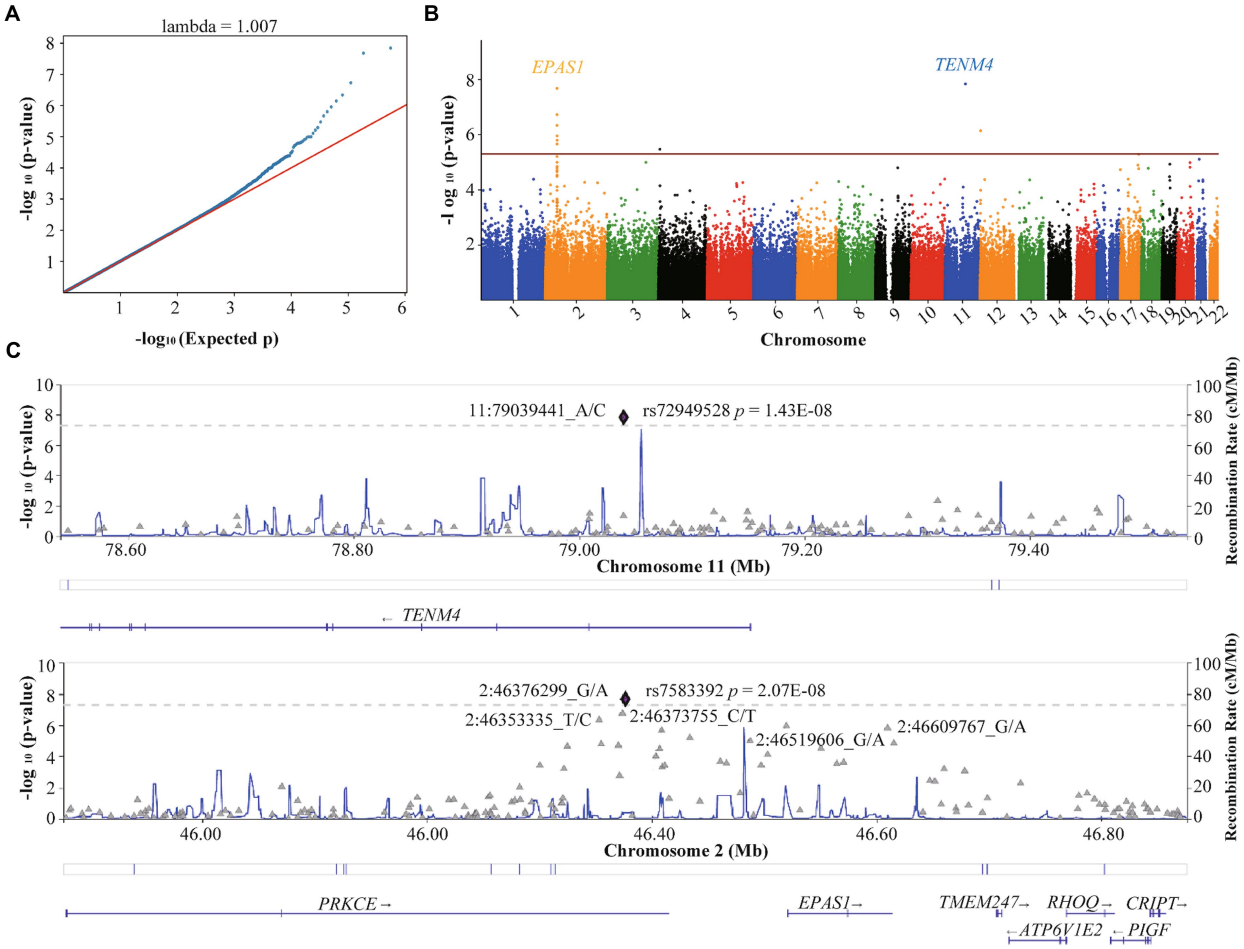
Quantile–quantile plot and Manhattan plot of the association analysis of high altitude. **(A)** Quantile–quantile plot. **(B)** Manhattan plot. The red line represents the genome-wide significance threshold *p* = 5E-05, and the blue line represents the genome-wide suggestiveness threshold *p* = 5E-06. **(C)** Regional plots of SNPs with threshold *p* < 5E-08 for high-altitude. The plots were generated using Locuszoom.

The QQ plot of GWAS analysis results related to HA revealed an expansion coefficient of 1.007 (Figure 3A), indicating no significant systematic bias in the correlation results. Furthermore, the Manhattan plot (Figure 3B) of GWAS analysis results related to HA showed that SNPs located on the *EPAS1* gene (member of the HIF gene family) on chromosome 2p21 exhibited the strongest association with HA. During the GWAS analysis of HA, we identified 39 SNPs that were significantly associated with HA (*p* < 5.0 × 10^−5^), as presented in Table 2. Notably, a consistent region on chromosome 2, including six significant SNPs in the *EPAS1* gene (rs4953342, rs1562453, rs7589621, rs1992846, rs12467821, and rs7583392), was found to be associated with HA. Of particular interest, rs7583392 in *EPAS1* (*p* = 2.07 × 10^−8^) and rs72949528 in *TENM4* (*p* = 1.43 × 10^−8^) surpassed the genome-wide significance threshold of 5.0 × 10^−8^ (Figure 3C).

**Table 2:** Association between SNPs and altitude from GWAS analysis.

SNP-ID	Chr	Position	Genes	Alleles		MAF	*p*-value
				A	B		
rs6662517	1	203294284	*LINC01353*; *LINC01136*	A	G	0.149	4.12E-05
rs4953342	2	46324908	*EPAS1*	G	A	0.144	2.19E-05
rs1562453	2	46353335	*EPAS1*	C	T	0.222	4.58E-07
rs7589621	2	46355243	*EPAS1*	A	G	0.176	1.61E-05
rs1992846	2	46370442	*EPAS1*	T	C	0.191	1.94E-05
rs12467821	2	46373755	*EPAS1*	T	C	0.215	1.86E-07
rs7583392	2	46376299	*EPAS1*	A	G	0.220	2.07E-08
rs1109286	2	46406791	*LOC124907762*	G	A	0.265	3.22E-05
rs13011481	2	46408509	*LOC124907762*	T	C	0.273	2.15E-06
rs12986899	2	46433613	*LINC02583*	A	C	0.288	6.13E-06
rs75768182	2	46487414	*TMEM247*; *ATP6V1E2*	A	G	0.318	1.00E-05
rs896210	2	46519606	*ATP6V1E2*	A	G	0.267	1.10E-06
rs11683396	2	46550554	*RHOQ*	T	C	0.201	2.90E-05
rs34544165	2	46609767	*PIGF*	A	G	0.381	1.58E-06
rs2242033	2	46615271	*PIGF*	A	G	0.374	1.40E-05
rs62268859	3	150071590	*TMEM183B*; *LOC105374313*	A	G	0.421	1.01E-05
rs4689431	4	6398051	*PPP2R2C*	T	C	0.133	3.35E-06
rs17064736	8	2114361	*MYOM2*	C	T	0.126	4.97E-05
rs1952349	9	86829027	*ZCCHC6*; *GAS1*	T	G	0.437	1.57E-05
rs11156566	10	131688957	*TCERG1L*; *LINC01164*	A	G	0.364	4.06E-05
rs72949528	11	79039441	*TENM4*	C	A	0.057	1.43E-08
rs111428991	12	3021260	*TEAD4*	G	A	0.067	7.14E-07
rs7964035	12	19113199	*CAPZA3*; *PLEKHA5*	A	G	0.309	4.20E-05
rs9570346	13	60719593	*LINC00378*; *MIR3169*	A	C	0.206	4.36E-05
rs17693812	17	959020	*NXN*	A	G	0.102	1.84E-05
rs1421133	17	31760587	*MIR365B*; *COPRS*	G	T	0.155	4.09E-05
rs72861370	17	69485813	*MAP2K6*	A	C	0.191	1.26E-05
rs7210086	17	72645559	*SLC39A11*	C	A	0.171	1.70E-05
rs17780256	17	72646784	*SLC39A11*	C	A	0.175	5.08E-06
rs470256	18	26713320	*PCAT18*; *AQP4*	C	T	0.365	1.64E-05
rs4805278	19	28845757	*LOC100420587*; *LINC00906*	T	C	0.376	3.32E-05
rs6510150	19	30361177	*ZNF536*	A	G	0.331	4.53E-05
rs1125867	19	30364744	*ZNF536*	G	A	0.340	1.18E-05
rs2274950	20	50277887	*PELATON*	C	A	0.478	1.50E-05
rs1984908	20	50302984	*LINC01270*	C	T	0.491	1.02E-05
rs2825641	21	19562938	*MIR548XHG*; *LINC01683*	G	A	0.065	4.69E-05
rs2017705	21	21508930	*NCAM2*	G	A	0.171	7.71E-06
rs1041831	21	35969641	*LOC101928269*	G	A	0.108	4.36E-05
rs2835226	21	36000386	*LOC101928269*	T	C	0.256	4.90E-05

SNP, single nucleotide polymorphism; Chr, chromosome; A, minor allele; B, major allele; MAF, minor allele frequency. *p* < 5 × 10^−5^ indicates genome-wide significance.

### 3.3 SNPs associated with HA-related body composition traits

**Figure 4:**
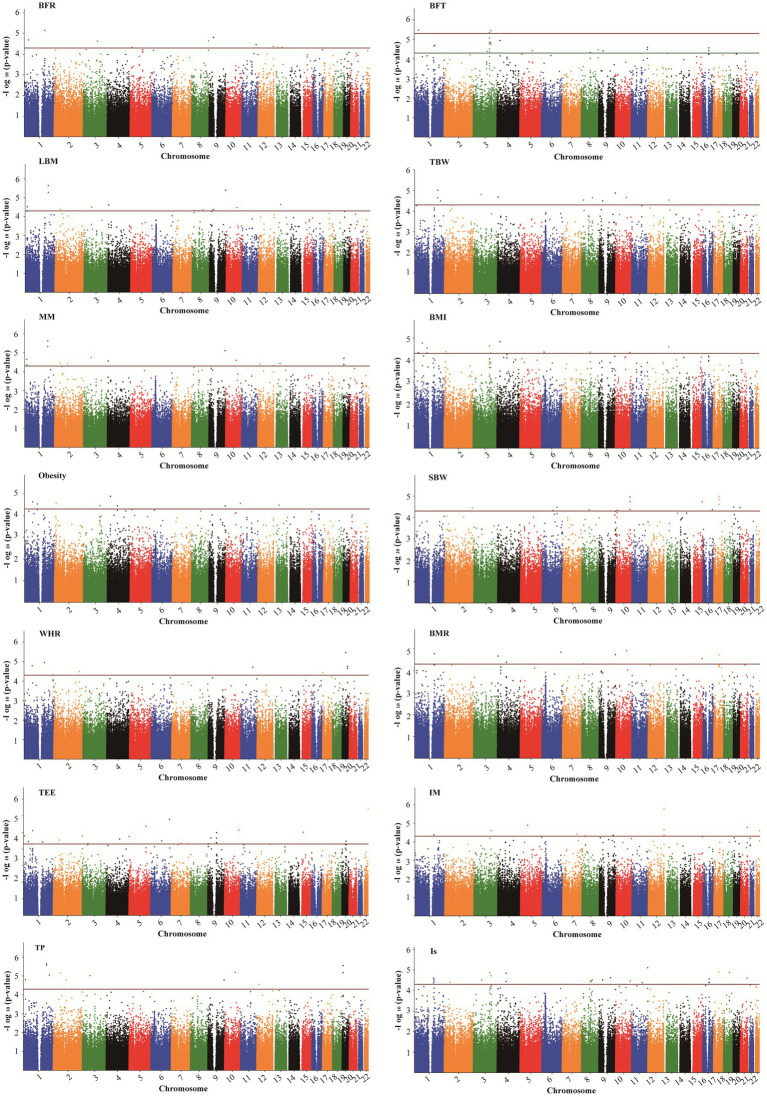
Manhattan plots showing association of all SNPs with body composition traits. SNPs are plotted on the *x*-axis according to their position on each chromosome against association with these traits on the *y*-axis (shown as – log10 *p*-value). The red dashed line shows genome-wise significance with a *p*-value threshold of 5E-05. BFR, body fat ratio; BFT, body fat; LBM, lean body mass; TBW, total body water; BMI, body mass index; SBW, standard weight; WHR, waist to hip ratio; BMR, basal metabolic rate; TEE, total energy expenditure; IM, impedance; TP, protein; Is, inorganic salt.

Supplementary Figure S1 displays the correlation between altitude and human body composition indicators. The findings revealed strong correlations (*r* > 0.6) among 8 body composition indicators (TBW, LBM, MM, SBW, BMR, TEE, TP, and Is). Additionally, a certain negative correlation exists between altitude and 9 body composition indicators (BFT, LBM, TBW, MM, SBW, BMR, TEE, TP, and Is). In the GWAS analysis of 7 HA-related body composition indicators (LBM, TBW, MM, SBW, BMR, TP, and Is), we identified significant associations with specific SNPs. Specifically, 14 SNPs were associated with LBM, 11 SNPs with TBW, 15 SNPs with MM, 16 SNPs with SBW, 9 SNPs with BMR, 12 SNPs with TP, and 26 SNPs with Is (*p* < 5.0 × 10^−5^). Table 3 and Figure 4 present these associations. Notably, two SNPs (rs77267056 in *RXRA* and rs4934485 near *PANK1*) were significantly correlated with LBM, TBW, MM, BMR, and TP (Table 3 and Figure 4).

**Table 3:** Results of GWAS analysis of seven altitude-related body composition indicators.

Traits	SNP-ID	Chr	Position	Genes	Alleles		MAF	*p*-value
					A	B		
LBM	rs7528206	1	18480623	*KLHDC7A*	C	T	0.098	3.04E-05
	rs35897870	1	18593057	*KLHDC7A;PAX7*	T	C	0.155	4.95E-05
	rs10801160	1	192914891	*RGS2;LINC01032RGS2;LINC01032*	A	G	0.416	2.28E-06
	rs10921285	1	192917652	*RGS2;LINC01032RGS2;LINC01032*	T	C	0.433	5.22E-06
	rs7577004	2	46793786	*SOCS5;LINC01118*	A	G	0.336	4.22E-05
	rs6762466	3	63362458	*SYNPR*	A	C	0.143	3.17E-05
	rs36027048	4	6741568	*BLOC1S4;KIAA0232*	C	T	0.475	2.41E-05
	rs1564425	8	20512665	*LZTS1-AS1;SNORD3F*	G	A	0.296	4.69E-05
	rs6471649	8	57753326	*LOC286178;LINC01602*	T	C	0.249	4.93E-05
	rs10504881	8	89761018	*RIPK2*	A	G	0.207	4.40E-05
	rs1633498	9	30115052	*LINGO2;LINC01242*	A	C	0.315	4.20E-05
	rs77267056	9	134425487	*RXRA*	T	C	0.109	4.03E-06
	rs4934485	10	89576163	*SLC16A12;PANK1SLC16A12;PANK1*	C	T	0.163	3.33E-05
	rs9316544	13	51434102	*INTS6*	T	C	0.229	2.25E-05
TBW	rs10801160	1	192914891	*RGS2;LINC01032RGS2;LINC01032*	A	G	0.414	9.69E-06
	rs10921285	1	192917652	*RGS2;LINC01032RGS2;LINC01032*	T	C	0.431	2.25E-05
	rs17042719	1	216517713	*ESRRG*	C	T	0.123	3.23E-05
	rs6762466	3	63362458	*SYNPR*	A	C	0.143	1.55E-05
	rs36027048	4	6741568	*BLOC1S4;KIAA0232*	C	T	0.473	2.09E-05
	rs352809	8	15764945	*TUSC3*	C	T	0.127	2.88E-05
	rs10504881	8	89761018	*RIPK2*	A	G	0.207	2.25E-05
	rs1633498	9	30115052	*LINGO2;LINC01242*	A	C	0.316	3.20E-05
	rs77267056	9	134425487	*RXRA*	T	C	0.109	1.32E-05
	rs4934485	10	89576163	*SLC16A12;PANK1SLC16A12;PANK1*	C	T	0.162	2.13E-05
	rs76574246	13	41452944	*OR7E37P;RGCC*	T	C	0.264	2.89E-05
MM	rs7528206	1	18480623	*KLHDC7A*	C	T	0.098	2.24E-05
	rs35897870	1	18593057	*KLHDC7A;PAX7*	T	C	0.154	4.33E-05
	rs10801160	1	192914891	*RGS2;LINC01032RGS2;LINC01032*	A	G	0.414	2.34E-06
	rs10921285	1	192917652	*RGS2;LINC01032RGS2;LINC01032*	T	C	0.431	4.69E-06
	rs7577004	2	46793786	*SOCS5;LINC01118*	A	G	0.338	3.65E-05
	rs35921849	2	109710857	*SOWAHC;RGPD6*	A	T	0.183	3.84E-05
	rs6762466	3	63362458	*SYNPR*	A	C	0.143	1.77E-05
	rs36027048	4	6741568	*BLOC1S4;KIAA0232*	C	T	0.473	2.71E-05
	rs77267056	9	134425487	*RXRA*	T	C	0.109	7.68E-06
	rs4934485	10	89576163	*SLC16A12;PANK1SLC16A12;PANK1*	C	T	0.162	2.53E-05
	rs1706613	12	17498784	*SKP1P2;LINC02378*	C	A	0.076	4.08E-05
	rs2030880	12	130992530	*ADGRD1*	T	C	0.132	4.72E-05
	rs9316544	13	51434102	*INTS6*	T	C	0.231	3.66E-05
	rs604625	19	7507640	*TEX45*	A	G	0.225	1.97E-05
	rs1133380	19	7508415	*TEX45*	T	C	0.226	4.13E-05
SBW	rs4972909	2	229657147	*DNER*	T	C	0.494	3.57E-05
	rs9351150	6	88382816	*CNR1;LOC101928936*	A	G	0.203	4.73E-05
	rs9401579	6	122724709	*PKIB*	G	A	0.412	3.35E-05
	rs55720422	8	61657898	*ASPH*	A	G	0.227	4.40E-05
	rs12264216	10	9965253	*LOC101928272;LOC101928298*	C	A	0.077	4.68E-05
	rs363225	10	117264991	*SLC18A2*	C	T	0.406	1.10E-05
	rs363238	10	117279248	*PDZD8*	A	C	0.280	1.78E-05
	rs12413507	10	117348747	*PDZD8*	T	C	0.295	4.16E-05
	rs10886063	10	117364506	*PDZD8*	A	G	0.332	4.13E-05
	rs7180301	15	95544299	*LINC00924;NR2F2-AS1*	T	C	0.203	1.85E-05
	rs376490	16	77871761	*VAT1L*	G	T	0.270	4.27E-05
	rs4309445	17	43609648	*DHX8*	A	G	0.370	1.49E-05
	rs1004357	17	43614158	*ETV4;MEOX1*	G	A	0.487	2.45E-05
	rs80294306	17	43650365	*MEOX1*	T	C	0.398	1.06E-05
	rs757527	19	1781084	*ATP8B3*	T	C	0.328	3.26E-05
	rs543052	19	55514027	*SSC5D*	A	C	0.153	3.49E-05
BMR	rs12564661	1	164445865	*LOC100422212;PBX1*	T	A	0.125	1.68E-05
	rs36027048	4	6741568	*BLOC1S4;KIAA0232*	C	T	0.473	2.15E-05
	rs6814880	4	74280047	*MTHFD2L*	G	A	0.211	4.12E-05
	rs6930928	6	156284422	*MIR1202;SNORD28B*	A	C	0.303	1.43E-05
	rs352809	8	15764945	*TUSC3*	C	T	0.127	4.93E-05
	rs77267056	9	134425487	*RXRA*	T	C	0.109	1.84E-05
	rs4934485	10	89576163	*SLC16A12;PANK1SLC16A12;PANK1*	C	T	0.162	1.23E-05
	rs7180301	15	95544299	*LINC00924;NR2F2-AS1*	T	C	0.203	2.85E-05
	rs1004357	17	43614158	*ETV4;MEOX1*	G	A	0.487	1.88E-05
TP	rs7528206	1	18480623	*KLHDC7A*	C	T	0.098	1.59E-05
	rs10801160	1	192914891	*RGS2;LINC01032RGS2;LINC01032*	A	G	0.414	2.29E-06
	rs10921285	1	192917652	*RGS2;LINC01032RGS2;LINC01032*	T	C	0.431	2.68E-06
	rs17042719	1	216517713	*ESRRG*	C	T	0.123	8.86E-06
	rs1473099	2	60222575	*LINC01793;MIR4432HG*	G	A	0.388	6.82E-06
	rs35921849	2	109710857	*SOWAHC;RGPD6*	A	T	0.183	1.66E-05
	rs6762466	3	63362458	*SYNPR*	A	C	0.143	9.55E-06
	rs77267056	9	134425487	*RXRA*	T	C	0.109	1.62E-05
	rs4934485	10	89576163	*SLC16A12;PANK1SLC16A12;PANK1*	C	T	0.162	6.38E-06
	rs1706613	12	17498784	*SKP1P2;LINC02378*	C	A	0.076	2.86E-05
	rs604625	19	7507640	*TEX45*	A	G	0.225	2.84E-06
	rs1133380	19	7508415	*TEX45*	T	C	0.226	6.64E-06
Is	rs73028938	1	159802745	*FCRL6*	G	A	0.134	4.34E-05
	rs75978343	1	159811637	*FCRL6*	A	G	0.136	2.46E-05
	rs525194	1	164333878	*LOC100422212;PBX1*	A	G	0.147	2.82E-05
	rs12564661	1	164445865	*LOC100422212;PBX1*	T	A	0.125	3.54E-05
	rs7606976	2	46787476	*SOCS5;LINC01118*	A	C	0.461	4.03E-05
	rs75126787	3	69197537	*FRMD4B*	C	T	0.143	3.08E-05
	rs9820485	3	136509724	*STAG1*	C	A	0.156	1.37E-05
	rs62274290	3	146926748	*LINC02010;ZIC4*	T	G	0.058	1.90E-05
	rs28644493	4	74262959	*MTHFD2L*	G	A	0.244	3.65E-05
	rs6814880	4	74280047	*MTHFD2L*	G	A	0.211	1.43E-05
	rs17405819	8	75894349	*HNF4G;LINC01111*	T	C	0.459	3.72E-05
	rs13248565	8	76210508	*HNF4G;LINC01111*	C	T	0.443	3.55E-05
	rs9298399	8	82976410	*LOC101927141;LINC01419*	C	A	0.160	3.39E-05
	rs10504881	8	89761018	*RIPK2*	A	G	0.207	3.00E-05
	rs1633498	9	30115052	*LINGO2;LINC01242*	A	C	0.316	3.03E-05
	rs10117181	9	96261175	*HSD17B3*	T	C	0.388	2.36E-05
	rs2083069	10	123119632	*ACADSB;HMX3ACADSB;HMX3*	C	T	0.215	3.47E-05
	rs6483414	11	88744046	*GRM5*	T	C	0.252	4.20E-05
	rs79369108	11	133751360	*OPCML;LOC646522*	T	C	0.235	7.76E-06
	rs11859517	16	53147335	*CHD9*	T	C	0.165	2.76E-05
	rs12598049	16	53282942	*CHD9*	G	A	0.163	3.94E-05
	rs8063660	16	53319247	*CHD9*	C	T	0.164	4.21E-05
	rs62049817	16	53336109	*CHD9;LOC643802*	C	T	0.163	3.94E-05
	rs12937489	17	43694223	*MEOX1;SOST*	T	C	0.221	1.26E-05
	rs4940376	18	48424968	*ZBTB7C;CTIF*	G	A	0.446	1.29E-05
	rs6127813	20	56679998	*TFAP2C;BMP7*	T	C	0.345	2.48E-05

SNP, single nucleotide polymorphism; Chr, chromosome; A, minor allele; B, major allele; MAF, minor allele frequency; LBM, lean body mass; TBW, total body water; MM, muscle mass; SBW, standard weight; BMR, basal metabolic rate; TP, total protein; Is, inorganic salt. p < 5 × 10^−5^ indicates genome-wide significance.

In addition, five SNPs [rs11588213 (*STK40*), rs12567152 (*PRRC2C*), rs1607960 (*LSAMP*), rs61136314, and rs2236293 (*TMEM8B*)] were found to be associated with both BFR and BFT. A total of 12 significant SNPs associated with BMI were identified, of which 7 SNPs were also significantly associated with obesity, including rs1337406 (*WLS*), rs673612 (*NTNG1*), rs4449107 (*FAM84A*), rs9820485 (*STAG1*), rs73247924 (*CCKAR*), rs2083069 (*ACADSB*), and rs4942190 (*DNAJC15*). GWAS analysis for WHR revealed that 6 SNPs associated with WHR (*p* < 5 × 10^−5^), among which rs1337406 in the *WLS* gene on chromosome 1 had the strongest correlation with WHR (*p* = 1.61 × 10^−6^). GWAS analysis for TEE showed that a total of 24 SNPs were associated with TEE, and rs62241230 (*p* = 3.92 × 10^−7^), rs6930928 (*p* = 1.65 × 10^−6^), and rs4912800 (*p* = 4.23 × 10^−6^) with significant *p* < 5 × 10^−6^ (Table 4).

**Table 4:** Results of GWAS analysis of other seven body composition indicators.

Traits	SNP-ID	Chr	Position	Genes	Alleles		MAF	*p*-value
					A	B		
BFR	rs11588213	1	36368877	*STK40*	A	G	0.050	2.03E-05
	rs12567152	1	171519379	*PRRC2C*	G	A	0.307	6.99E-06
	rs1607960	3	117082892	*LSAMP*	T	C	0.466	2.32E-05
	rs13360149	5	16730761	*MYO10*	A	C	0.151	4.70E-05
	rs61136314	8	141077651	*PTK2;DENND3*	T	C	0.117	2.28E-05
	rs2236293	9	35841786	*TMEM8B*	A	G	0.241	1.53E-05
	rs2115645	11	119378778	*USP2*	G	A	0.124	3.41E-05
	rs80212198	12	126332697	*LINC02359;LOC283435*	T	C	0.179	4.21E-05
	rs1999421	13	30960825	*TEX26*	C	T	0.246	4.90E-05
	rs10161776	13	65115876	*LINC00355;LINC01052*	T	C	0.401	4.81E-05
BFT	rs11588213	1	36368877	*STK40*	A	G	0.050	3.36E-06
	rs57346682	1	164776132	*PBX1*	A	G	0.062	2.19E-05
	rs12567152	1	171519379	*PRRC2C*	G	A	0.307	1.99E-05
	rs1607960	3	117082892	*LSAMP*	T	C	0.466	4.15E-05
	rs34894639	3	136079816	*PPP2R3A*	T	C	0.145	1.54E-05
	rs645040	3	136207780	*MSL2;PCCB*	G	T	0.148	1.63E-05
	rs548288	3	136250913	*PCCB*	T	C	0.154	1.36E-05
	rs483465	3	136329135	*PCCB*	A	G	0.162	8.42E-06
	rs667920	3	136350630	*STAG1*	G	T	0.164	4.39E-05
	rs9820485	3	136509724	*STAG1*	C	A	0.156	4.45E-06
	rs7621025	3	136553404	*STAG1*	T	C	0.163	3.09E-05
	rs62274290	3	146926748	*LINC02010;ZIC4*	T	G	0.058	3.53E-06
	rs17564921	3	147171868	*LINC02010;ZIC4*	C	A	0.070	1.86E-05
	rs62275291	3	147247599	*LINC02010;ZIC4*	T	C	0.060	1.53E-05
	rs4605637	4	25651434	*SLC34A2*	C	T	0.222	1.15E-05
	rs7701167	5	104816620	*NUDT12;RAB9BP1*	T	C	0.117	3.85E-05
	rs17405819	8	75894349	*HNF4G;LINC01111*	T	C	0.459	4.70E-05
	rs35359188	8	76432651	*LINC01111*	A	G	0.257	4.76E-05
	rs61136314	8	141077651	*PTK2;DENND3*	T	C	0.117	3.43E-05
	rs2236293	9	35841786	*TMEM8B*	A	G	0.241	3.88E-05
	rs73038693	11	133738100	*OPCML;LOC646522*	G	A	0.268	3.30E-05
	rs79369108	11	133751360	*OPCML;LOC646522*	T	C	0.235	2.57E-05
	rs11859517	16	53147335	*CHD9*	T	C	0.165	3.71E-05
	rs7204230	16	53158419	*CHD9*	C	T	0.209	2.72E-05
BMI	rs11588213	1	36368877	*STK40*	A	G	0.050	4.53E-05
	rs1337406	1	68108170	*WLS*	G	A	0.434	1.68E-05
	rs673612	1	107412584	*NTNG1*	C	T	0.089	2.83E-05
	rs17018946	1	107413504	*NTNG1*	G	A	0.092	4.78E-05
	rs4449107	2	14499792	*LINC00276;FAM84A*	A	G	0.489	4.15E-05
	rs9820485	3	136509724	*STAG1*	C	A	0.156	2.31E-05
	rs62274290	3	146926748	*LINC02010;ZIC4*	T	G	0.058	4.77E-05
	rs73247924	4	26510638	*CCKAR;TBC1D19*	A	G	0.170	1.45E-05
	rs7739578	6	21160151	*CDKAL1*	A	G	0.228	4.34E-05
	rs35359188	8	76432651	*LINC01111*	A	G	0.257	4.37E-05
	rs2083069	10	123119632	*ACADSB;HMX3ACADSB;HMX3*	C	T	0.215	4.59E-05
	rs4942190	13	43086793	*DNAJC15*	T	G	0.270	2.50E-05
Obesity	rs1337406	1	68108170	*WLS*	G	A	0.434	2.36E-05
	rs673612	1	107412584	*NTNG1*	C	T	0.089	2.91E-05
	rs4449107	2	14499792	*LINC00276;FAM84A*	A	G	0.489	2.72E-05
	rs9820485	3	136509724	*STAG1*	C	A	0.156	3.47E-05
	rs73247924	4	26510638	*CCKAR;TBC1D19*	A	G	0.170	1.29E-05
	rs13147116	4	84038487	*LOC101928978*	T	C	0.355	3.59E-05
	rs10858322	9	135046005	*FCN1;OLFM1*	C	T	0.414	3.63E-05
	rs2083069	10	123119632	*ACADSB;HMX3ACADSB;HMX3*	C	T	0.215	2.76E-05
	rs4942190	13	43086793	*DNAJC15*	T	G	0.270	3.33E-05
WHR	rs1337406	1	68108170	*WLS*	G	A	0.432	1.61E-05
	rs12567152	1	171519379	*PRRC2C*	G	A	0.307	1.11E-05
	rs12746625	1	242130817	*PLD5*	T	C	0.080	4.85E-05
	rs36037305	2	210819425	*CPS1;ERBB4*	G	A	0.261	3.16E-05
	rs7107215	11	99945185	*CNTN5*	C	A	0.059	1.98E-05
	rs7221022	17	878293	*NXN*	C	T	0.146	3.73E-05
	rs7245985	19	30219503	*URI1;ZNF536*	G	T	0.179	3.44E-06
	rs17658470	19	44612083	*CEACAM22P*	T	C	0.387	1.75E-05
	rs10409208	19	44837471	*BCAM;NECTIN2*	C	T	0.293	2.21E-05
TEE	rs6695721	1	9466667	*LOC100506022;SLC25A33*	G	A	0.119	1.62E-05
	rs841407	1	43058596	*SLC2A1-AS1;FAM183A*	C	T	0.152	3.57E-05
	rs77971268	1	78758314	*IFI44;ADGRL4*	T	C	0.057	7.78E-06
	rs4657042	1	161512730	*FCGR2A*	T	C	0.198	3.72E-05
	rs11897143	2	50318386	*NRXN1*	T	C	0.309	2.89E-05
	rs155841	3	1435325	*CNTN6;CNTN4*	G	A	0.201	1.64E-05
	rs13086717	3	46098007	*XCR1;CCR1*	G	A	0.313	4.77E-05
	rs404950	4	113165086	*ANK2*	G	A	0.424	2.49E-05
	rs6879627	5	2109787	*CTD-2194D22.4;LOC100506858*	T	C	0.445	1.80E-05
	rs4912800	5	142078130	*GNPDA1;NDFIP1*	A	G	0.319	4.23E-06
	rs464339	6	90492476	*MIR4464;MAP3K7*	G	A	0.469	3.20E-05
	rs6930928	6	156284422	*MIR1202;SNORD28B*	A	C	0.303	1.65E-06
	rs3801721	7	82173592	*CACNA2D1*	T	G	0.213	4.53E-05
	rs6980105	7	143884414	*TCAF1*	A	G	0.498	4.88E-05
	rs10758574	9	4209925	*GLIS3*	T	C	0.460	4.89E-05
	rs10967078	9	25799668	*LINC01241*	G	T	0.230	2.16E-05
	rs10746883	9	71646656	*TRPM3;TMEM2*	C	T	0.488	4.21E-05
	rs7031916	9	73323159	*ANXA1;LOC101927358*	G	A	0.108	4.22E-05
	rs17058804	9	73368049	*ANXA1;LOC101927358*	C	T	0.108	2.22E-05
	rs75705077	9	73464276	*ANXA1;LOC101927358*	A	G	0.100	1.03E-05
	rs2420679	10	120276084	*MIR4682;RPL21*	A	G	0.330	7.18E-06
	rs10444861	15	33753502	*RYR3*	C	T	0.306	9.80E-06
	rs11666907	19	34915935	*LINC01838;ZNF30-AS1*	A	C	0.345	3.42E-05
	rs62241230	22	50316037	*DENND6B*	T	C	0.189	3.92E-07
IM	rs55788686	1	158008158	*KIRREL1*	A	G	0.193	4.24E-05
	rs73161649	3	143270749	*SLC9A9*	A	G	0.188	2.44E-05
	rs9292179	5	58832796	*RAB3C*	T	C	0.159	1.28E-05
	rs4727878	7	118572016	*ANKRD7;LINC02476*	A	G	0.355	3.79E-05
	rs7821268	8	29606921	*DUSP4;LINC00589*	C	T	0.253	4.66E-05
	rs10817815	9	115688741	*DEC1;LOC101928775*	C	T	0.387	4.43E-05
	rs2030880	12	130992530	*ADGRD1*	T	C	0.132	1.73E-06
	rs4759547	12	131143498	*ADGRD1;LINC01257*	G	T	0.374	4.43E-05
	rs10773849	12	131145078	*ADGRD1;LINC01257*	G	T	0.342	2.18E-05
	rs6092186	20	55834075	*LINC01441;CBLN4*	C	T	0.369	1.65E-05
	rs13056610	22	47588203	LINC01644;LINC00898	G	A	0.064	2.52E-05

SNP, single nucleotide polymorphism; Chr, chromosome; A, minor allele; B, major allele; MAF, minor allele frequency; BFR, body fat percentage; BFT, body fat; BMI, body mass index; WHR, waist to hip ratio; TEE, total energy expenditure; IM, impedance. *p* < 5 × 10^−5^ indicates genome-wide significance.

### 3.4 SNPs further screening by LASSO

**Figure 5:**
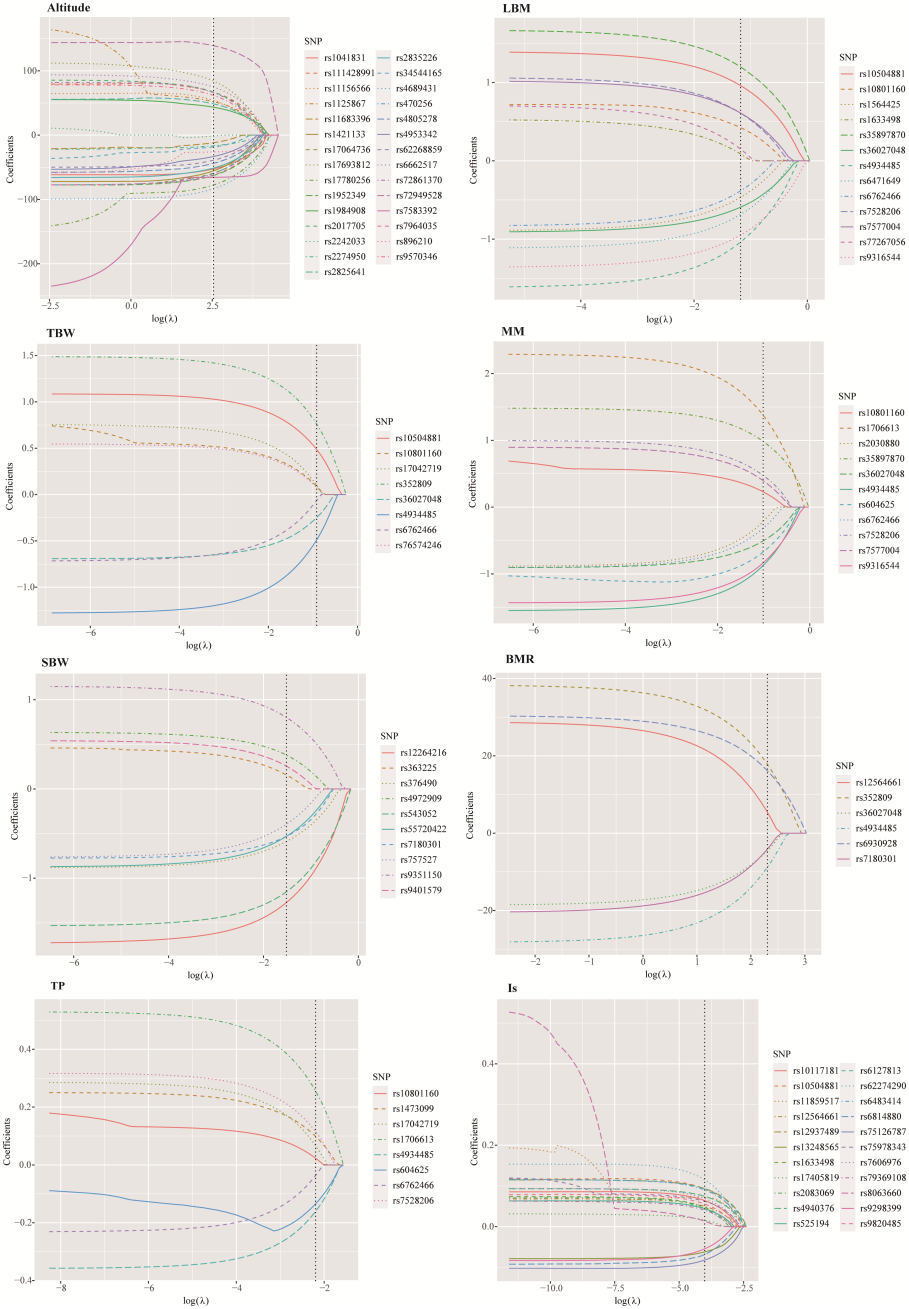
SNPs selection using the least absolute shrinkage and selection operator.

The LASSO method was employed to further screen the most significant loci associated with 7 HA-related human body component indicators. The optimal lambda (λ) parameters in the LASSO regression model were selected through 10-fold cross validation. The LASSO coefficient profiles of the SNPs with non-zero coefficients were determined by the optimal lambda (λ) (Supplementary Figure S2). There are two dashed lines in the cross-validation diagram, one is the input value with the minimum mean squared deviation and the other is the input value of the minimum mean squared error. We take the geometric mean of the two as the λ value. As shown in the Figure 5, the 39 SNPs associated with HA were reduced to 29 according to the LASSO regression method when *λ* = 12.65. When *λ* = 10.04, the 9 SNPs associated with BMR were reduced to 6. When *λ* = 0.018, the number of SNPs associated with IS decreased from 26 to 22. When *λ* = 0.31, the number of SNPs associated with LBM decreased from 14 to 13. When *λ* = 0.36, the number of SNPs associated with muscle mass decreased from 15 to 11. When *λ* = 0.22, the number of SNPs associated with SBW decreased from 16 to 10. When *λ* = 0.40, the number of SNPs associated with TBW decreased from 11 to 8 (Supplementary Table S2).

## 4 Discussion

In order to adapt to the extreme anoxic environment of the plateau region, the Indigenous people of Tibet have developed a markedly different set of physiological characteristics. During prolonged hypoxia, it can affect a person’s body composition, such as reductions in body weight, fat free mass (FFM), MM, and TBW. The study aims to provide a preliminary basis for discovering the role of genetic factors in the changes in HA-related body composition in Tibetan populations adapted to HA environment. From the 279,608 imputed SNPs and 14 body composition phenotypes investigated, we found that 39 SNPs were significantly associated with HA and 103 SNPs were significantly associated with 7 HA-related body composition phenotypes (LBM, TBW, MM, SBW, BMR, TP, and Is) (*p* < 5 × 10^−5^). Of these, 14 SNPs were located in genes with known functions, helping to explain the genetic and physiological mechanisms that lead to changes in body composition in HA populations.

In this study, we found a negative correlation between 9 body compositions (BFT, LBM, TBW, MM, SBW, BMR, TEE, TP, and Is) and HA, and 7 indicators (LBM, TBW, MM, SBW, BMR, TP, and Is) showed significant differences in different altitude stratification in the Tibetan college students. Similarly, these indicators have previously been reported to decrease with increasing altitude (7, 8, 20). In addition, inverse association between obesity and altitude has previously been reported (21). Although the association between BMI, WHR, obesity, and HA was not significant in this study, these indicators showed a negative correlation trend with altitude.

We identified 39 SNPs related to HA, 6 SNPs (rs4953342, rs1562453, rs7589621, rs1992846, rs12467821, and rs7583392) of which were located in *EPAS1* gene on chromosome 2. The *EPAS1* gene encodes hypoxia inducible factor 2α (HIF-2α), a transcription factor that is involved in the induction of oxygen-regulatory genes when oxygen levels decline. As one of the major gene in the HIF pathway, *EPAS1* has been reported as the most important candidate gene for HA adaptation (22, 23). Adaptive mutations in *EPAS1* may serve as an adaptive strategy in HA indigenous peoples. Bhandari et al. (24) showed that individuals carrying the derived alleles of rs12467821 in *EPAS1* has lower hemoglobin levels than wild-type allele carriers in in Tibetans and Sherpas. The SNP rs1562453 has been confirmed to be associated with the susceptibility to high altitude pulmonary hypertension (HAPH) in Chinese Han population (25). For rs7589621, linear-by-linear association test revealed a significant increasing trend of major G allele and genotype GG frequencies with increasing altitude among native Tibetans (26). In our study, we found a possible involvement of a novel SNP (rs7583392) in *EPAS1* that was associated with HA.

In the early stage, we observed a significant decrease in LBM as altitude increased. GWAS analysis revealed that rs77267056 in *RXRA* was associated with LBM. *RXRA* acts as a transcription factor for various nuclear receptors, including *PPARα*, and is known to play a crucial role in fatty acid metabolism. Previous research has shown that under hypoxic conditions, the activity of the PPARα/RXRA complex is reduced, leading to a suppression of fatty acid metabolism (27). Prolonged hypoxia can alter DNA methylation patterns. Studies have demonstrated that CpG island methylation in the promoter region of *RXRA* is lower at HA compared to low altitudes, potentially resulting in increased expression (28). *RXRA*, a member of the retinoic acid receptor family, is essential for normal hematopoietic function during development, and its methylation levels are positively correlated with hemoglobin levels (29). These findings suggest that variants of *RXRA* may be involved in HA adaptations.

In summary, we have identified several regions on the chromosome associated with HA human body components, some of which are consistent with previously reported SNPs. It is worth noting that some SNPs related to traits are found in the intergenic regions of functional coding genes (30). Studies have shown that a large number of disease-related SNPs are found in the non-coding RNAs lacking conservation, known as lincRNAs, which has become an area of interest (31). For instance, polymorphisms of *LincPINT* and *Linc00599* have been found to be associated with HAPE susceptibility in the Chinese population (32). However, there are limited reports on SNPs in lincRNAs associated with HA-related body components. In our study, we discovered that rs10801160 and rs10921285, located 22 kb downstream of lncRNA *RP11-139E24.1*, are associated with HA-related traits such as LBM, MM, and TP. Currently, there are no reported studies on the adaptation of lncRNA *RP11* to hypoxia in HA. Most studies have focused on investigating the role of the lncRNA *RP11* gene in cancer. However, it has been discovered that hypoxia-induced lncRNA *RP11-367G18.1* regulates hypoxia-induced target genes by regulating histone markers of H4K16Ac. This regulation leads to epithelial-mesenchymal transition, metastasis, and tumorigenicity (33). In our study, we observed significant associations between HA-related characteristics such as LBM, MM, and TP with two specific variants, rs10801160 and rs10921285, in the lncRNA *RP11-139E24.1* gene.

Furthermore, previous studies have observed that rs645040 near *MSL2* is significantly associated with lipid traits, such as triglycerides (34) and high-density lipoprotein (HDL) cholesterol (35). Moreover, the SNP rs7621025 (*STAG1*) was identified as a pleiotropic variant for HDL-cholesterol (36). In this study, we identified two loci (rs645040 and rs7621025) associated with BFT levels in the Tibetan college students. However, the identified loci related to other human body composition indicators in this study have not been reported yet. Therefore, further validation of the results of this study is needed. Furthermore, the results of this study have a positive impact on public health, especially for people living in high-altitude areas. Firstly, by analyzing genetic variations and body composition indicators related to high-altitude adaptability, we can better understand the physiological adaptation process of Tibetan college students to high-altitude environments. This helps to develop personalized health management and prevention measures to improve their quality of life in high-altitude environments. Secondly, the research results can provide a scientific basis for public health policies in high-altitude areas. By understanding the performance of different body components in high-altitude adaptation, governments and health institutions can develop more effective health policies to meet the special health needs of residents in high-altitude areas. Lastly, our results also provide a reference for similar research in other high-altitude areas, promoting the development of global high-altitude health research.

This study has several limitations. Firstly, it only included samples of Tibetan college students from HA areas, which may result in insufficient representativeness and restrict the generalizability of the findings to the entire Tibetan population or other populations in different regions. Secondly, the possibility of genetic heterogeneity between Tibetan populations in HA areas and populations in other regions could potentially impact the relationship between genes and human body composition. Thirdly, variations in environmental conditions between HA areas and other regions may influence human body composition and introduce confounding factors that could complicate the association between genes and human body composition. Lastly, it is important to note that GWAS research can solely identify associations between genes and human components, and cannot establish specific functional mechanisms. Therefore, further experimental research is warranted to elucidate these associations.

In conclusion, several candidate loci associated with HA and HA-related body composition indicators, such as LBM, TBW, MM, BMR, SBW, TP and Is were identified. Additionally, it was found that some loci or genes were common across these traits, suggesting a shared genetic basis. These differential loci indicate a strong early genetic adaptation to life at high altitude, followed by the spread of these adaptive populations. Further studies are required to gain a deeper understanding of the underlying mechanisms through functional investigations.

## Data availability statement

The raw data supporting the conclusions of this article will be made available by the authors, without undue reservation.

## Ethics statement

The studies involving humans were approved by the Ethics Committee of the Medical College of Xizang Minzu University (No. 20180–18). The studies were conducted in accordance with the local legislation and institutional requirements. The participants provided their written informed consent to participate in this study.

## Author contributions

XGL: Writing – original draft, Methodology. SX: Writing – original draft, Methodology, Data curation. XML: Writing – original draft, Software, Data curation. YW: Writing – review & editing, Investigation, Data curation. YS: Writing – review & editing, Project administration. HZ: Writing – review & editing, Methodology, Investigation. WY: Writing – original draft, Investigation. DY: Writing – review & editing, Project administration. TJ: Writing – original draft, Conceptualization. XH: Writing – original draft, Conceptualization.

## Footnotes

^1^ http://https://www.amap.com/

^2^ https://www.weaoo.com/changdu-181248.html

^3^ https://jingweidu.bmcx.com/

^4^ http://locuszoom.org/
