# Supplementary figures and images for: Novel insight into the genetic signatures of altitude adaptation related body composition in Tibetans
Source: Front Public Health. 2024 May 14;12:1355659. doi: 10.3389/fpubh.2024.1355659 (PMC11130355; doi:10.3389/fpubh.2024.1355659)

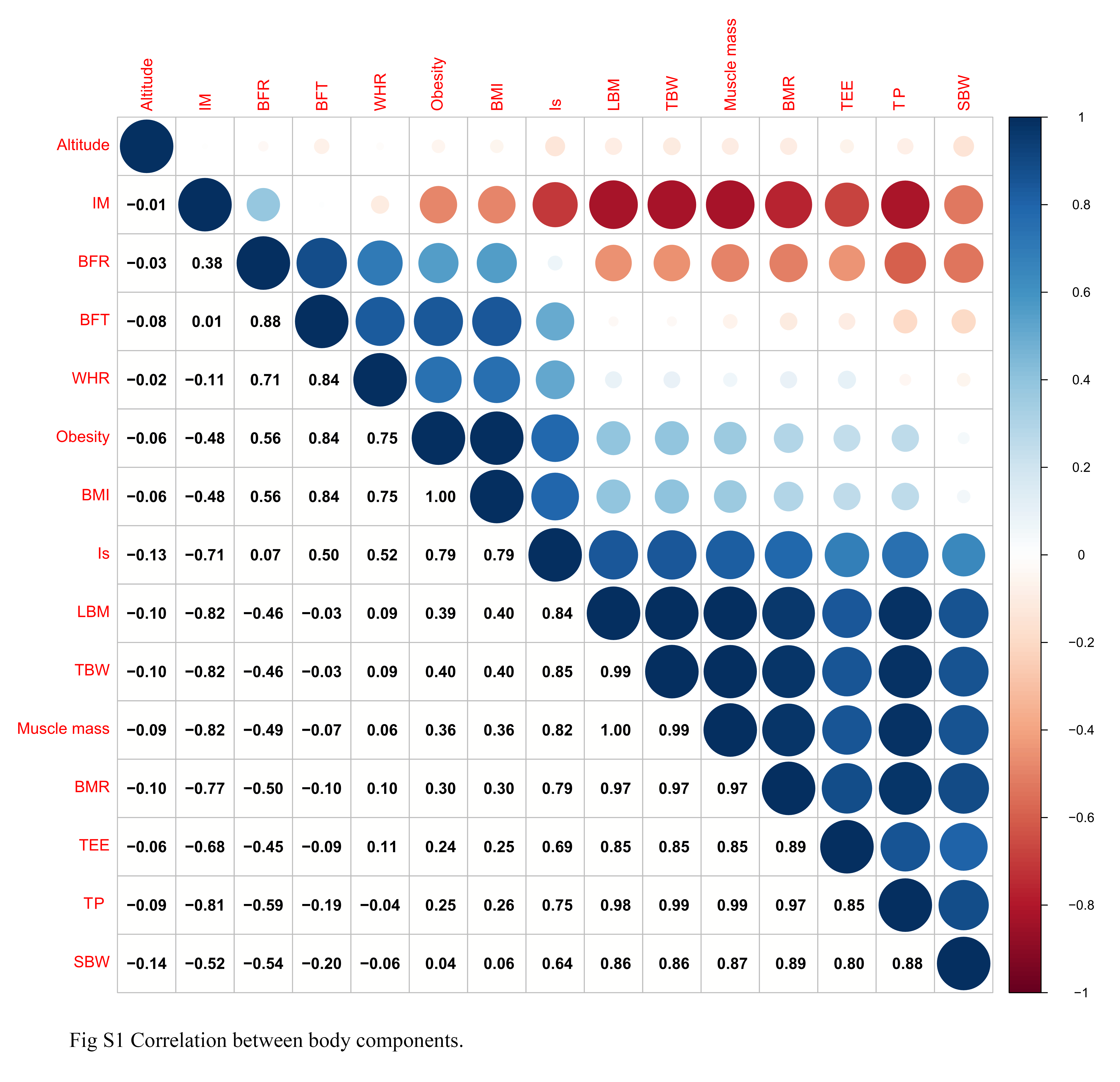

Supplement: Supplementary file 1 [file Image_1.TIF]

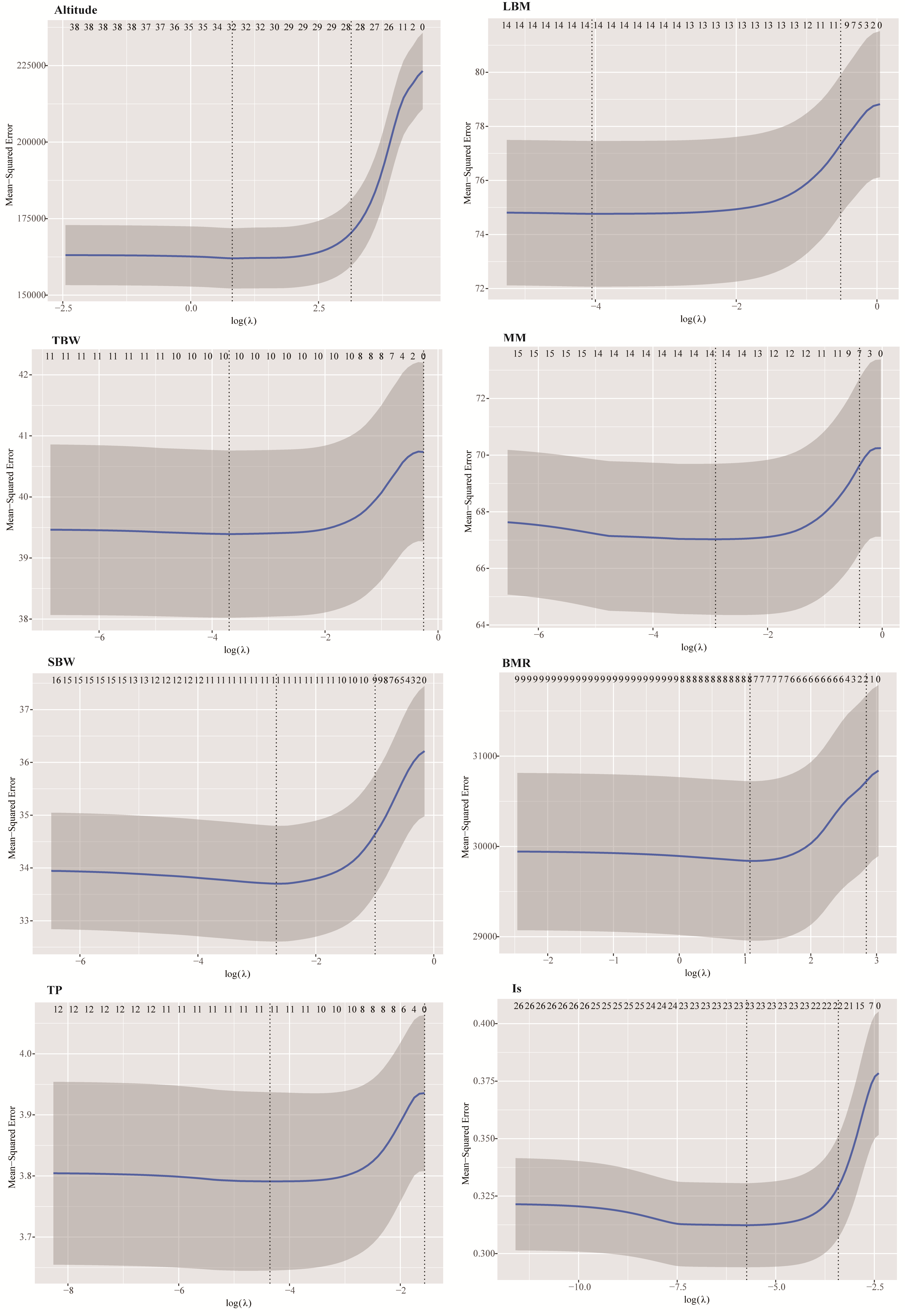

Supplement: Supplementary file 2 [file Image_2.TIF]
