# Supplementary material for: Novel insight into the genetic signatures of altitude adaptation related body composition in Tibetans
Source: Front Public Health. 2024 May 14;12:1355659. doi: 10.3389/fpubh.2024.1355659 (PMC11130355; doi:10.3389/fpubh.2024.1355659)
Supplement: Supplementary file 3 [file Table_1.DOCX]

**Table S1. Summary of samples collected**

| Traits | Cases | | |
| --- | --- | --- | --- |
|  | Valid (n) | Missing (n) | Mean ± SD |
| Age | 994 | 0 | 18.50 ± 1.18 |
| Altitude | 994 | 0 | 3882.00 ± 447.45 |
| Air pressure | 994 | 0 | 30.09 ±1.09 |
| BFR | 994 | 0 | 23.47 ± 8.36 |
| BFT | 994 | 0 | 14.42 ± 6.43 |
| LBM | 986 | 8 | 46.42 ± 8.83 |
| TBW | 994 | 0 | 33.45 ± 6.36 |
| MM | 994 | 0 | 42.92 ± 8.35 |
| BMI | 994 | 0 | 21.73 ± 3.12 |
| Obesity | 994 | 0 | -0.81 ± 14.28 |
| SBW | 994 | 0 | 61.33 ± 6.01 |
| WHR | 964 | 30 | 0.77 ± 0.06 |
| BMR | 994 | 0 | 1390.95 ± 175.28 |
| TEE | 994 | 0 | 2059.09 ± 295.89 |
| IM | 994 | 0 | 518.32 ± 82.84 |
| TP | 994 | 0 | 9.5 ± 1.98 |
| Is | 994 | 0 | 3.51 ± 0.61 |
| 1500-2500m | 11 | 0 | / |
| 2500-3500m | 106 | 0 | / |
| 3500-4500m | 742 | 0 | / |
| 4500-5500m | 135 | 0 | / |

SD, standard deviation; BFR, body fat ratio; BFT, body fat; LBM, lean body mass; TBW, total body water; MM, muscle mass; BMI, body mass index; SBW, standard weight; WHR, waist to hip ratio; BMR, Basal metabolic rate; TEE, total energy expenditure; IM, impedance; TP, total protein; Is, inorganic salt

**Table S2. Summary of GWAS and LASSO for selected altitude and altitude-related trait variants in the study.**

| Traits | No. of variants (n) | | SNP-ID (Lasso) |
| --- | --- | --- | --- |
|  | GWAS results (p < 5×10-5) | Lasso selection |  |
| Altitude | 39 | 29 | rs1041831, rs111428991, rs11156566, rs1125867, rs11683396, rs1421133, rs17064736, rs17693812, rs17780256, rs1952349, rs1984908, rs2017705, rs2242033, rs2274950, rs2825641, rs2835226, rs34544165, rs4689431, rs470256, rs4805278, rs4953342, rs62268859, rs6662517, rs72861370, rs72949528, rs7583392, rs7964035, rs896210, and rs9570346 |
| BFR | 10 | 10 | rs11588213, rs12567152, rs1607960, rs13360149, rs61136314, rs2236293, rs2115645, rs80212198, rs1999421, and rs10161776 |
| BFT | 24 | 18 | rs11588213, rs11859517, rs12567152, rs1607960, rs17405819, rs17564921, rs2236293, rs35359188, rs4605637, rs57346682, rs61136314, rs62274290, rs645040, rs7204230, rs73038693, rs7701167, rs79369108, and rs9820485 |
| LBM | 14 | 13 | rs10504881, rs10801160, rs1564425, rs1633498, rs35897870, rs36027048, rs4934485, rs6471649, rs6762466, rs7528206, rs7577004, rs77267056, and rs9316544 |
| TBW | 11 | 8 | rs10504881, rs10801160, rs17042719, rs352809, rs36027048, rs4934485, rs6762466, and rs76574246 |
| MM | 15 | 11 | rs10801160, rs1706613, rs2030880, rs35897870, rs36027048, rs4934485, rs604625, rs6762466, rs7528206, rs7577004, and rs9316544 |
| BMI | 12 | 11 | rs11588213, rs1337406, rs2083069, rs35359188, rs4449107, rs4942190, rs62274290, rs673612, rs73247924, rs7739578, and rs9820485 |
| Obesity | 9 | 9 | rs10858322, rs13147116, rs1337406, rs2083069, rs4449107, rs4942190, rs673612, rs73247924, and rs9820485 |
| SBW | 16 | 10 | rs12264216, rs363225, rs376490, rs4972909, rs543052, rs55720422, rs7180301, rs757527, rs9351150, and rs9401579 |
| WHR | 9 | 9 | rs10409208, rs12567152, rs12746625, rs1337406, rs17658470, rs36037305, rs7107215, rs7221022, rs7245985, and rs9401579 |
| BMR | 9 | 6 | rs12564661, rs352809, rs36027048, rs4934485, rs6930928, and rs7180301 |
| TEE | 24 | 20 | rs10444861, rs10746883, rs10967078, rs11897143, rs13086717, rs155841, rs17058804, rs2420679, rs404950, rs464339, rs4657042, rs4912800, rs62241230, rs6695721, rs6930928, rs6980105, rs7031916, rs75705077, rs77971268, and rs841407 |
| IM | 11 | 10 | rs10773849, rs10817815, rs13056610, rs2030880, rs4727878, rs55788686, rs6092186, rs73161649, rs7821268, and rs9292179 |
| Pr | 12 | 8 | rs10801160, rs1473099, rs17042719, rs1706613, rs4934485, rs604625, rs6762466, and rs7528206 |
| Is | 26 | 22 | rs10117181, rs10504881, rs11859517, rs12564661, rs12937489, rs13248565, rs1633498, rs17405819, rs2083069, rs4940376, rs525194,rs6127813, rs62274290, rs6483414, rs6814880, rs75126787, rs75978343, rs7606976, rs79369108, rs8063660, rs9298399, and rs9820485 |

BFR, body fat ratio; BFT, body fat; LBM, lean body mass; TBW, total body water; MM: muscle mass; BMI, body mass index; SBW, standard weight; WHR, waist to hip ratio; BMR, Basal metabolic rate; TEE, total energy expenditure; IM, impedance; Pr, protein; Is, inorganic salt
